# Supplementary material for: Ultrafast kinetics of the antiferromagnetic-ferromagnetic phase transition in FeRh
Source: Nat Commun. 2022 May 30;13:2998. doi: 10.1038/s41467-022-30591-2 (PMC9151753; doi:10.1038/s41467-022-30591-2)
Supplement: Supplementary file 1 — Supplementary Information [file 41467_2022_30591_MOESM1_ESM.pdf]

(1) Double Pump Lock-In Detection

To correctly interpret the signal by two pumps at the lock-in amplifier we first indicate all possible signals under action of the pump pulses and the lock-in amplifier (the schematic illustration is shown in Fig. S1). We distinguish three signals,

$$V_1(t) = A_1 \sin(\omega_1 t + \theta_1), \tag{1}$$

$$V_2(t) = A_2 \sin(\omega_2 t + \theta_2) + A_{12} \sin(\omega_1 t + \theta_1), \tag{2}$$

$$V_r(t) = A_r \sin(\omega_r t + \theta_r). \tag{3}$$

$V_{1(2)}$ is the signal under action of pump 1(2) with an amplitude $A_{1(2)}$ at a repetition frequency of $\omega_{1(2)}$ and phase $\theta_{1(2)}$. In the case of V_2, it consists of the sum of the signal from pump 2 alone and the combined signal from the coupling between pump 1 and 2, if pump 1 arrives to the sample before pump 2. Here, A_2 is the signal amplitude from pump 2 at the repetition frequency of ω_2 and phase θ_2 and A_{12} is the signal amplitude under action of pump 2 and modulated by pump 1 at the repetition frequency ω_1 and phase θ_1. V_r is the internal reference signal of the lock-in amplifier where ω_r can be fixed as a higher harmonic provided by the external function generator and θ_r is the phase of the reference signal. The repetition frequencies are set so that $\omega_1 = \omega_r$ where $\omega_r/2\pi$ is 500 Hz and $\omega_2 = 2\omega_1$. The lock-in amplifier then uses a phase-sensitive detector to multiply the sum of all signals with the internal reference signal,

$$V_{PSD} = A_1 A_r \sin(\omega_r t + \theta_1) \sin(\omega_r t + \theta_r) + A_2 A_r \sin(2\omega_r t + \theta_2) \sin(\omega_r t + \theta_r) + A_{12} A_r \sin(\omega_r t + \theta_1) \sin(\omega_r t + \theta_r). \tag{4}$$

Solving the product of the pump induced signals with the reference signal results in two DC terms and three AC terms,

$$V_{PSD} = \frac{1}{2} A_r \big[A_1[\cos(\theta_1 - \theta_r) - \cos(2\omega_r t + \theta_1 + \theta_r)] + A_2[\cos(\omega_r t + \theta_2 - \theta_r) - \cos(3\omega_r t + \theta_2 + \theta_r)] + A_{12}[\cos(\theta_1 - \theta_r) - \cos(2\omega_r t + \theta_1 + \theta_r)]\big]. \tag{5}$$

Then the phase-sensitive detector output is passed through a low pass filter where it suppresses any AC signals. This only leaves the two DC terms as shown below

$$V_{PSD} = \frac{1}{2} A_r \cos(\theta_1 - \theta_r)\, [A_1 + A_{12}], \tag{6}$$

$$V_{PSD} \propto [A_1 + A_{12}]. \tag{7}$$

The reference phase in eq. (6) can be tuned such that $\cos(\theta_1 - \theta_r)$ can be set to 1. Then the signal detected by the lock-in amplifier is proportional to the amplitude A_1 of pump 1 and the coupled pump 1 and 2 amplitude A_{12} as is shown in eq. (7). A_1 refers to the THz emission under action of pump 1. If pump 1 arrives before pump 2 then A_{12} refers to a pump 1-induced modulation of the signal under action of pump 2. It is clear that A_{12} is proportional to the change in THz emission under action of pump 2 (ΔE) with and without pump 1, respectively.

It is then easy to exclude the signal of A_1 by either subtracting the THz emission signal where pump 2 is blocked or by positioning the chopper in the beam path of pump 2 which means that the frequencies ω_1 and ω_2 are switched. In our case we chose the latter method.

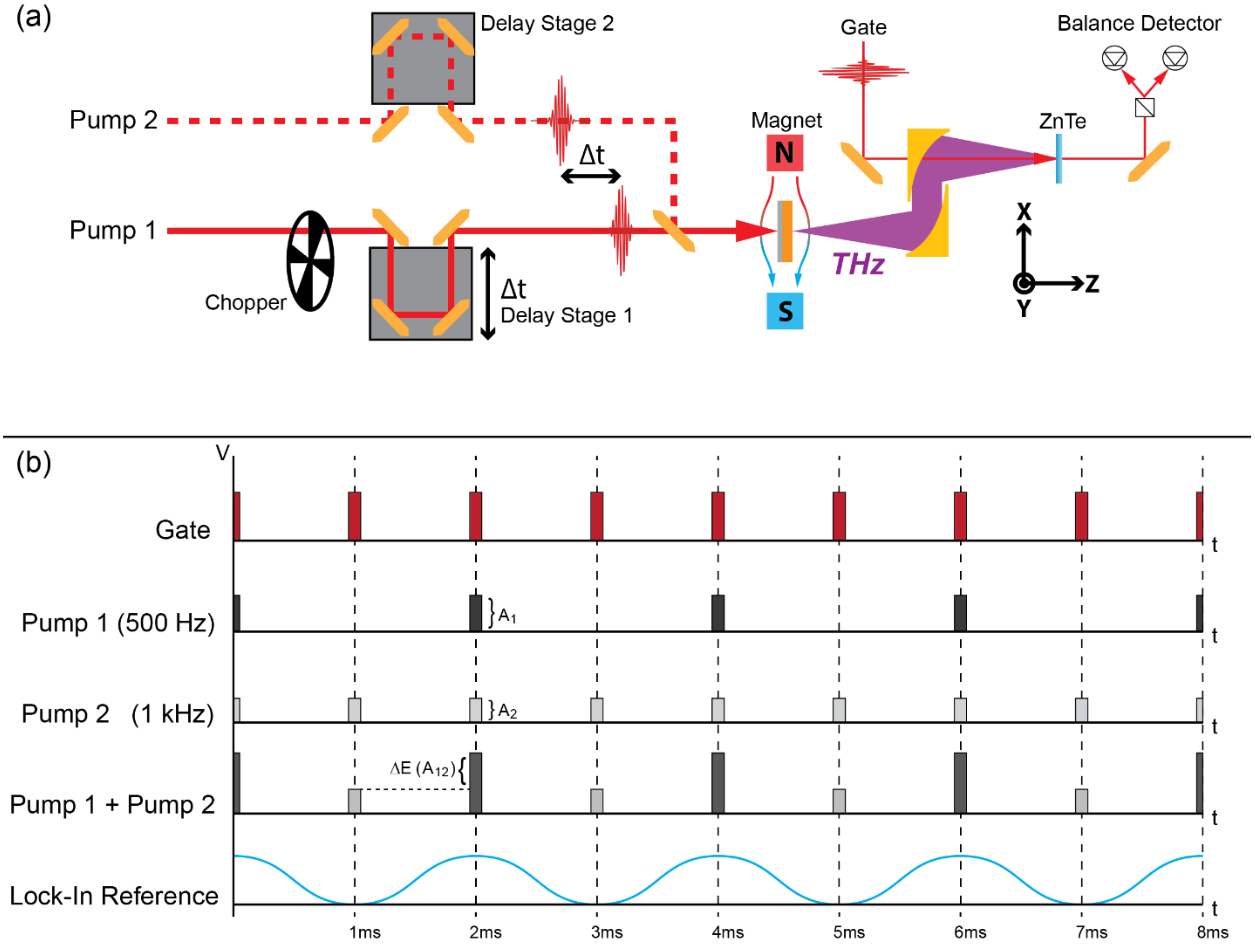

Figure S1 *(a) The principles of double pump THz emission. The delay stage (1) of pump 1 defines the time delay Δt and the delay stage (2) of pump 2 is fixed to set the temporal overlap with the gate pulse. The chopper is set at pump 1. (b) Signal processing of THz detection in the double pump THz emission spectroscopy. Voltages (y-axis) versus time (x-axis) generated at the detector due to excitation with different pump combinations. The gate pulse signal is set at 1 kHz. The THz signal* A_1 *is triggered by pump 1 at a repetition rate of 500 Hz while the THz signal* A_2 *is triggered by pump 2 at a repetition rate is 1 kHz, respectively. The change in THz emission under action of pump 2 with and without pump 1,* ΔE *(*A_{12}*), is therefore at 500 Hz. The lock-in reference frequency is set at 500 Hz.*

(2) Double pump THz emission signal at $t < 35$ ps

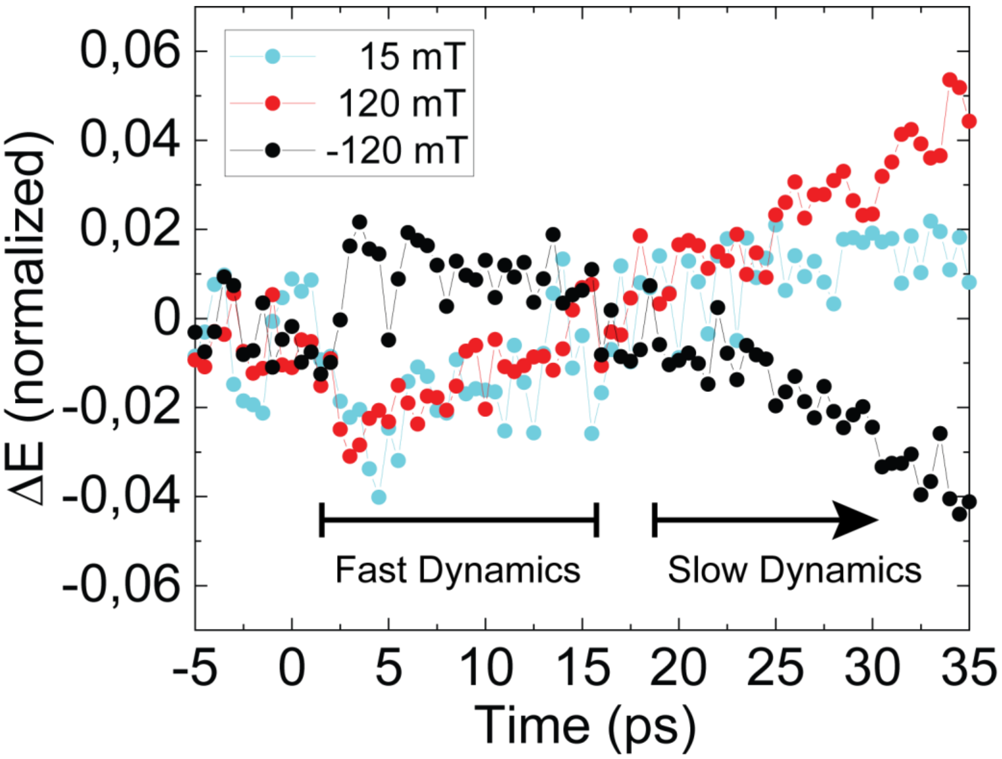

Figure S2 *The double pump THz emission due to emerging ferromagnetism in antiferromagnetic FeRh/Pt at applied field strengths $\mu_0 H$ = 15, 120 and -120 mT.*

(3) Magnetic field hysteresis

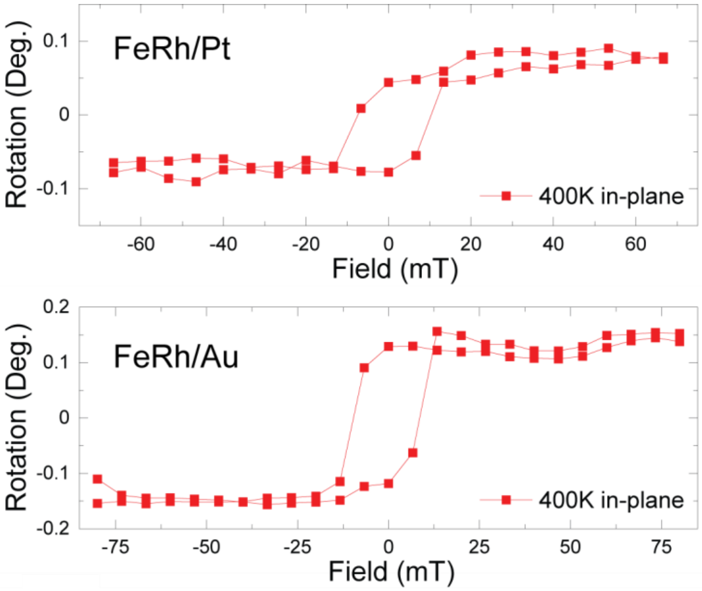

Figure S3 *Static MOKE experiments were performed on FeRh/Pt and FeRh/Au at 400K in an in-plane magnetic field setup. We observed no out-of-plane magnetic field hysteresis at 400K. We also observed no hysteresis at temperatures below the phase transition.*

(4) Normalizing the ΔE signal

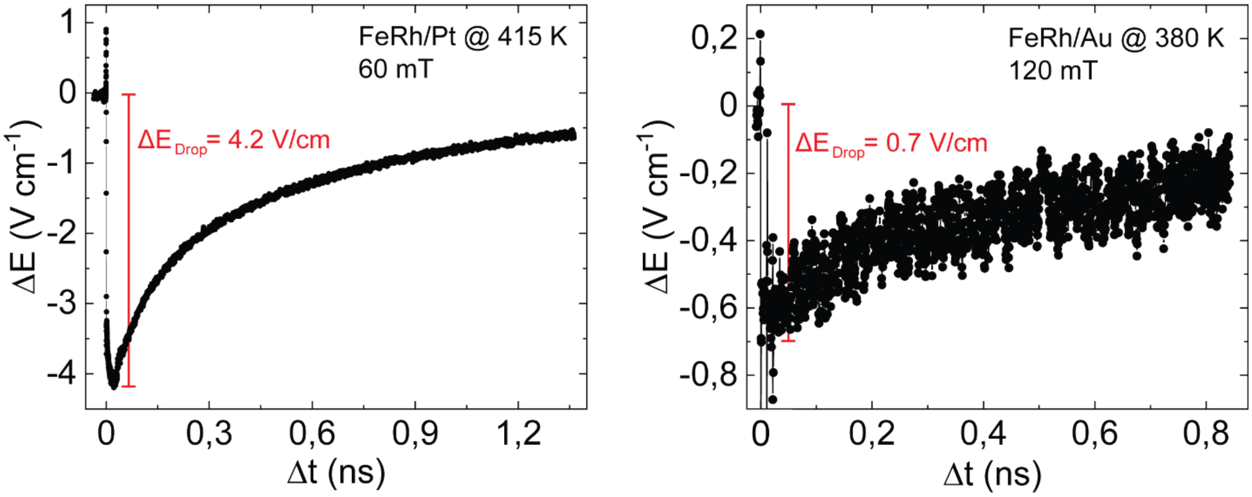

Figure S4 *The ΔE signal of FeRh in the ferromagnetic phase. (Left) FeRh/Pt measured at 415 K and 60 mT. (Right) FeRh/Au measured at 380 K at 120 mT. The* ΔE_{Drop} *value is the largest modulation of the THz field emitted from the ferromagnetic phase.*

In order to get an estimate for the laser-induced ferromagnetic (FM) fraction we normalize the ΔE signal in Figs. 5(a) and 7(a) by their respective ΔE_{Drop} signal, shown in Fig. S4. Here we assume that the ΔE_{Drop} value gives a good representation of the THz emission signal in a 100% ferromagnetic phase. The table below gives the maximum reached laser-induced FM fraction starting at the antiferromagnetic phase at 300 K. Here the FM fraction represents only the ferromagnetic domains where the magnetization is along the applied magnetic field and 1 is a fully ferromagnetic phase which is defined by the ΔE_{Drop}.

Field Strength (mT)	FeRh/Pt (FM fraction)	FeRh/Au (FM fraction)
30	0.33	
45	0.51	
60	0.62	0.47
75	0.68	
90	0.72	
105	0.75	
120		0.76

(5) Fitting procedure

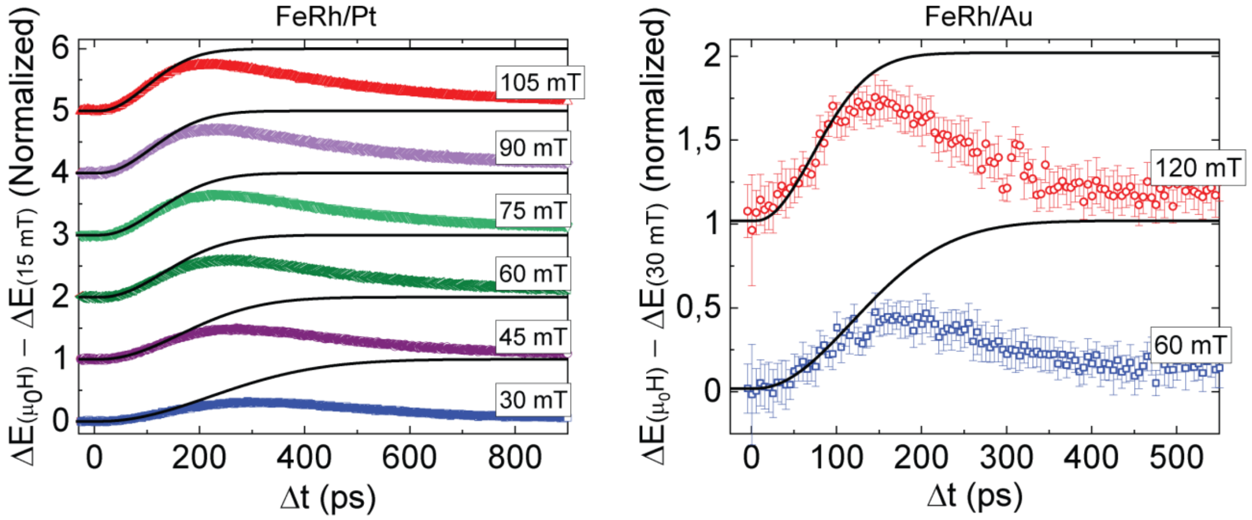

Figure S5 *(Left) The double-pump THz signal for various applied field strengths. (Right) the double-pump THz signal for the applied field of 120 and 60 mT. The solid lines are the fits to eq. (8). Only the data points at* $t < 100$ *ps were used to fit the formula. And the fit function is extrapolated beyond 100 ps with the extracted fit values.*

The data was fitted using the function,

$$f(t) = \mathcal{H}(t - t_{latency})\left(1 - e^{-K(t-t_{latency})^2}\right), \tag{8}$$

Here K is the growth parameter which correlates to the speed of the emerging ΔE signal. $\mathcal{H}(t)$ is the Heaviside-step-function and $t_{latency}$ is the latency.

Since the fit function does not take into account the decrease of the THz signal as a result of heat diffusing out of FeRh, we only fitted the data before 100 ps where the rise of the ΔE signal has not yet slowed down. Figure S5 shows the extrapolation of the fits above 100 ps. It shows the kinetics and timescale of the phase transition to a full ferromagnetic phase if there were no heat diffusion processes.

(6) Simulation of various exchange constants

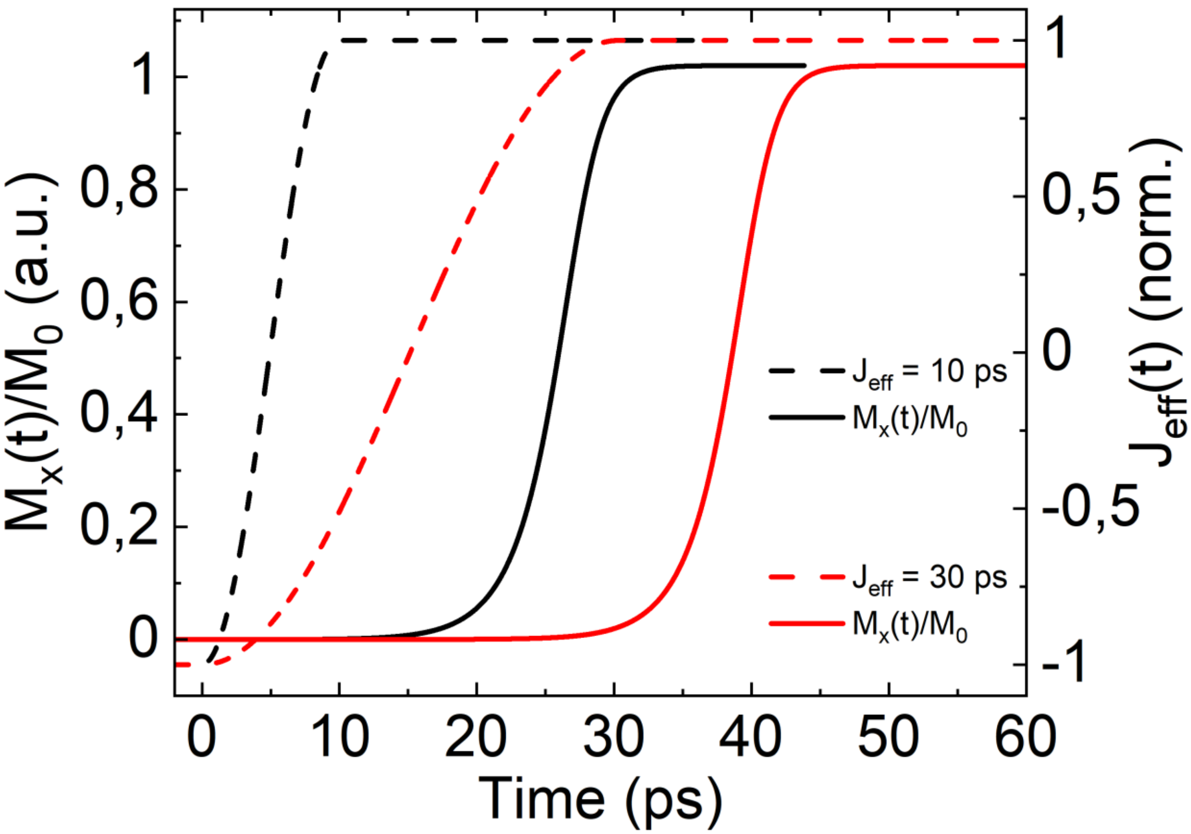

Figure S6 *The net magnetization $M(t)$ using a model that mimics the behavior of FeRh using 2 macrospins is simulated at a fixed applied magnetic field for the cases where the exchange constant $J_{eff}(t)$ changes at the timescale of 10 and 30 ps. The magnetization $M(t)$ as a result of the exchange constant $J_{eff}(t)$ change within 10 ps and 30 ps are indicated by black and red solid lines, respectively. The exchange constant $J_{eff}(t)$ are indicated by the dashed lines.*

(7) Pump 1 and 2 fluence dependence

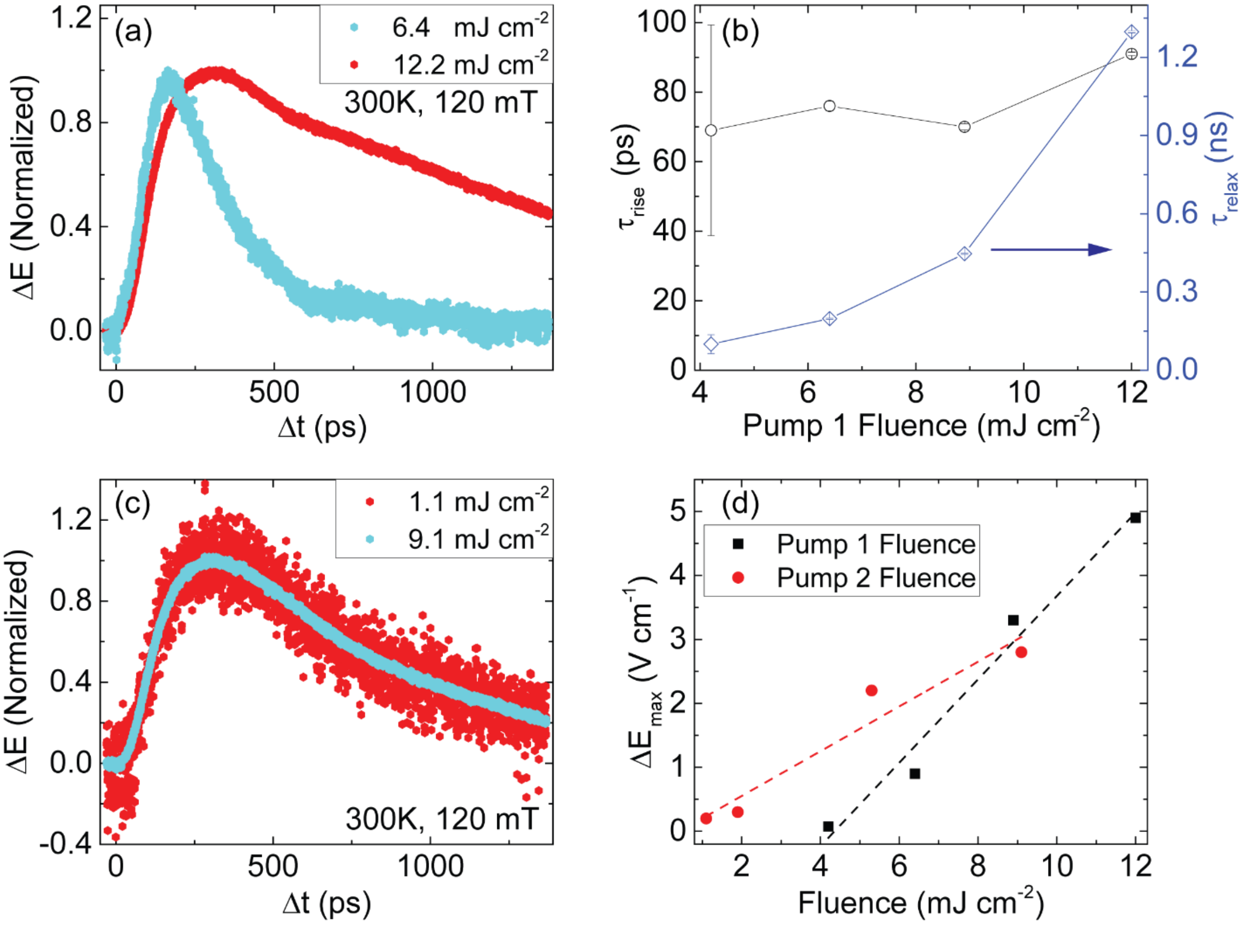

Figure S7 *The double pump THz emission signal where (a) the fluence of pump 1 is varied while pump 2 is fixed or (c) where the fluence of pump 1 is fixed while pump 2 is varied. The THz signal is normalized with respect to its own maximum peak signal. The ΔE signal of (a) was fitted with the function* $A_1\left(1-e^{-\frac{t}{\tau_{Rise}}}\right)+A_2e^{-\frac{t}{\tau_{Relax}}}$ *and the extracted* τ_{Rise} *and* τ_{Relax} *as a function of the pump 1 fluence is shown in (b). (d) The maximum ΔE signal as a function of the fluence of the pump 1 (black) or pump 2 (red). The measurement was done by varying the fluence of one pump while the fluence of the other pump was fixed at the maximum fluence.*

(8) Estimated electric and magnetic dipole THz emission

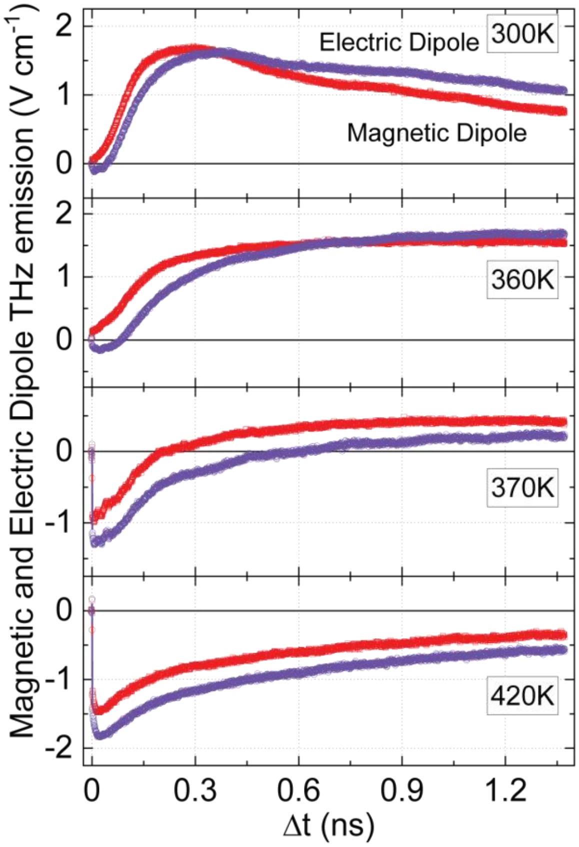

Figure S8 Electric and magnetic dipole THz emission. *The estimated dynamics of the magnetic dipole (red) and electric dipole (purple) contributions to the THz emission deduced from the data in Fig. 4 of the main text.*

When differentiating the magnetic- and electric-dipole sources we assume that they follow the net magnetization and are not affected by reflection and absorption in the multilayer. Measurements of THz waveforms pumped from the Pt- and MgO-sides, respectively, on a similar sample (see Ref. [12, N. Supplementary figure 2] in the reference list) show that the THz waveforms are similar. This means that absorption and reflection in the MgO-substrate and Pt-film and interfaces did not influence the THz waveforms and the temporal differences due to possible non-equivalent spatial profiles are negligible.

To estimate the dynamics of the THz emission from the electric- and magnetic-dipole source, we take the sum and difference between the ΔE signals for pumping from the MgO- (ΔE^{MgO}) and the Pt-side (ΔE^{Pt}): $E^{\mathrm{MD}} = \frac{\Delta E^{\mathrm{Pt}} + \Delta E^{\mathrm{MgO}}}{2}$ and $E^{\mathrm{ED}} = \frac{\Delta E^{\mathrm{MgO}} - \Delta E^{\mathrm{Pt}}}{2}$. When ΔE^{Pt} changes sign (see top panel of Fig. 4 of the main text), the THz emission from the magnetic- and electric-

dipole sources must be equally strong which cancel each other out. We use this as a reference point to normalize the ΔE^{MgO} THz signal:

$$\Delta E_{(t)}^{\mathrm{MgO/norm}} = \mathrm{sgn}(T_{\mathrm{PT}} - T) \times \frac{\Delta E_{(t)}^{\mathrm{MgO}}}{\mathrm{Max}(\left|\Delta E_{(t)}^{\mathrm{MgO}}\right|)} \times \Delta E_{(t=x)}^{\mathrm{MgO}}. \quad (9)$$

The normalized signal, $\Delta E_{(t)}^{\mathrm{MgO/norm}}$, is calculated by first dividing the signal $\Delta E_{(t)}^{\mathrm{MgO}}$ pumped from the MgO-side with respect to its maximum absolute value, $\mathrm{Max}(\left|\Delta E_{(t)}^{\mathrm{MgO}}\right|)$. The timestamp $t = x$ is defined as the time where the magnetic- and electric-dipole THz emissions are equally strong. For the case below T_{PT}, these sources interfere destructively and the timestamp is the point where $\Delta E_{(t)}^{\mathrm{Pt}}$ changes sign. Above T_{PT}, the sources interfere constructively and the timestamp corresponds to the point where $\Delta E_{(t)}^{\mathrm{MgO}}$ reaches its highest absolute value. The sign function accounts for the sign change of $\Delta E_{(t)}^{\mathrm{MgO}}$ at T_{PT}.
